# A Case of Poisoned Wound

**Published:** 1872-10

**Authors:** G. C. Paoli

**Affiliations:** Chicago


Article IV.—*A Case of Poisoned Wound.* By G. C. Paoli, M.D., Chicago.

I was called the eighth of July, in the afternoon, to see a boy four years of age, who previous to my arrival had been playing in the yard. On examination I found in the middle of the right leg a small elevation on the skin, which is called a papula. Neither swelling nor heat were perceptible, but the skin around the papula was very sensitive to the touch. Every attempt to stand on the leg produced the most excruciating pains. After careful examination, I concluded that my patient had been stung by some kind of an insect, but none of his playmates of the same age could give me any information that might corroborate my conjectures. I prescribed for the night a weak solution of permanganate of potassa, as an external application. Two hours after I had arrived at my residence, I was recalled, and informed that the patient was suffering from an inguinal hernia. To satisfy the parents I called again later in the evening, and found there was no rupture as they feared, but that one of the inguinal glands was somewhat swollen, as an effect of the irritation produced by the poisoned wound. We often find that small wounds or ulcerations in the lower extremities occasionally produce the above effects. The next morning on visiting my patient I found the following symptoms: The whole limb was swollen, and the temperature of the skin increased; pulse 130. He had been vomiting; tongue slightly coated; bowels constipated; urine scanty; very restless and sleepless. The whole limb was very sensitive to the touch. I ordered an emollient poultice, and, internally, carbonate of ammonia. In the evening I gave him an injection to move his bowels, and codeia to produce sleep.

The third day, pulse the same; the tongue dry and coated; had dozed a few hours; bowels moved. The fourth day, erysipelas appeared on the whole limb, involving the subcutaneous tissue. After the application of iodine, the erysipelas was dispersed about the twelfth day, but on the fourteenth day, a similar swelling and erysipelas also appeared, involving the whole left limb. During his illness he was several days delirious, and rejected all kinds of food except cold milk.

*Remarks on the case.*—About the eighth day after he was taken sick, I was informed that a gentleman living in the house saw a spider of brownish color with a yellow tint crawling on my patient's leg, and instantly killed it. According to the description, I supposed it was a species of spider called tarantula, which is by no means rare about Chicago, as I learn from a German physician named D. Helmuth, who has devoted much time to the study of insects. There have been the most exaggerated descriptions of the nervous symptoms caused by the bite of the Italian tarantula. We find the symptoms of chorea precisely similar, but in this case the nervous symptoms were entirely absent. I must add that the poison was a long time lingering in the system, and it took my little patient six weeks before he could walk and regain his strength.

